# Lhermitte’s sign and vitamin B12 deficiency: case report

**DOI:** 10.1590/S1516-31802009000300011

**Published:** 2009-10-06

**Authors:** Hélio Afonso Ghizoni Teive, Salo Haratz, Jorge Zavala, Renato Puppi Munhoz, Rosana Hermínia Scola, Lineu César Werneck

**Affiliations:** 1 MD, PhD. Associate professor, Neurology Service, Department of Internal Medicine, Hospital das Clínicas, Universidade Federal do Paraná (UFPR), Curitiba, Paraná, Brazil.; 2 MD. Resident physician, Neurology Service, Department of Internal Medicine, Hospital das Clínicas, Universidade Federal do Paraná (UFPR), Curitiba, Paraná, Brazil.; 3 MD. Neurologist, Neurology Service, Department of Internal Medicine, Hospital das Clínicas, Universidade Federal do Paraná (UFPR), Curitiba, Paraná, Brazil.; 4 MD, PhD. Head of Neurology Service, Department of Internal Medicine, Hospital das Clínicas, Universidade Federal do Paraná (UFPR), Curitiba, Paraná, Brazil.

**Keywords:** Myelopathy, Vitamin B12 deficiency, Ataxia, Aged, Polyneuropathies., Mielopatia, Deficiência de vitamina B12, Ataxia, Idoso, Polineuropatias.

## Abstract

**CONTEXT AND OBJECTIVE:**

Lhermitte’s sign, a classical neurological sign, is a rare manifestation of vitamin B12 deficiency. The aim here was to report on a case of an elderly patient with vitamin B12 deficiency whose first clinical manifestation was the presence of Lhermitte’s sign.

**CASE REPORT:**

We describe an elderly patient with vitamin B12 deficiency who presented cognitive dysfunction, peripheral polyneuropathy and sensory ataxia, and whose first clinical manifestation was the presence of Lhermitte’s sign. This sign is one of the rarest manifestations of vitamin B12 deficiency.

## RESUMO

**CONTEXTO E OBJETIVO:**

O sinal de Lhermitte, um sinal neurológico clássico, é uma rara manifestação clínica da deficiência de vitamina B12. O objetivo foi relatar o caso de um paciente idoso com deficiência de vitamina B12, cuja manifestação clínica inicial foi a presença do sinal de Lhermitte.

**RELATO DO CASO:**

Paciente idoso com deficiência de vitamina B12, apresentou-se com disfunção cognitiva, polineuropatia periférica e ataxia sensitiva, cuja manifestação clínica inicial foi a presença do sinal de Lhermitte. Este sinal é uma das mais raras manifestações da deficiência de vitamina B12.

## INTRODUCTION

Vitamin B12 deficiency is found in 15% of the geriatric population. The manifestations are hematological, gastrointestinal and neuropsychiatric.^1^^,^^2^ The neurological manifestation consists of subacute combined degeneration of the spinal cord that is clinically characterized by sensory ataxia, peripheral neuropathy, cognitive dysfunction and optic neuropathy.^1^^,^^2^

Lhermitte’s sign, also known as the barber’s shop sign, is more properly a symptom than a sign, and is described by patients as an electric-like sensation induced by forward flexion of the head.^3^^,^^4^ It was originally described by Marie and Chatelin in 1917, and one year later by Babinski and Dubois. The importance of this phenomenon was defined by the classical description by Jean L. Lhermitte in 1924 on a case of multiple sclerosis.^5^^,^^6^^,^^7^^,^^8^ One patient described it as follows: “When I bent my head, I felt a violent shock in my neck and a pain like an electric current running through my whole body, from the neck down the vertebral column into the feet”.^8^

The presence of Lhermitte’s sign is a rare manifestation.^3^^,^^4^^,^^5^^,^^6^^,^^7^^,^^8^ To estimate the validity and frequency of this sign for diagnosing myelopathy, we conducted a systematic review of the literature, including the following databases: DotLib - OVID; DotLib - OVID (Lippincott); EBSCO Biomedical Reference Collection; EBSCO - MedicLatina; EBSCO - Medical Literature Analysis and Retrieval System Online (Medline); ProQuest - Medical Library; PubMed; Cochrane Library; and Literatura Latino-Americana e do Caribe em Ciências da Saúde (Lilacs). Using the Medical Subject Headings/Descritores em Ciências da Saúde (MeSH/DeCS) terminology (Lhermitte AND Sign) we found, as shown in Table 1, that most studies reported to date are either in the form of case reports or demonstrate several procedural biases that may distort the accuracy values.^9^^,^^10^^,^^11^^,^^12^^,^^13^^,^^14^^,^^15^^,^^16^^,^^17^^,^^18^ Only two studies measured the diagnostic accuracy of Lhermitte’s sign, and they found that its sensitivity is markedly poor, ranging from 3 to 17%.^19^^,^^20^ One of these studies also found that it presented good specificity (97%) for nonspecific compressive myelopathy.^20^

We describe an elderly patient with vitamin B12 deficiency who initially presented with Lhermitte’s sign.

**Table 1.** Published articles on Lhermitte’s sign

Database	Search strategy	Results
DotLib - OVID	Lhermitte AND sign	3 reviews; 78 case series/reports
DotLib - OVID (Lippincott)	Lhermitte AND sign	4 reviews; 78 case series/reports
EBSCO - Biomedical Reference Collection	Lhermitte AND sign	1 review; 37 case series/reports
EBSCO - MedicLatina	Lhermitte AND sinal	6 case reports
EBSCO - Medline	Lhermitte [MeSH] AND sign [MeSH]	52 reviews; 945 case series/reports
ProQuest - Medical Library	Lhermitte [MeSH] AND sign [MeSH]	2 reviews; 42 case series/reports
PubMed	Lhermitte [MeSH] AND sign [MeSH]	52 reviews; 830 case series/reports
Cochrane	Lhermitte [MeSH] AND sign [MeSH]	2 reviews
Lilacs	Lhermitte [DeCS] AND sinal [DeCS]	20 case series/reports

Medline = Medical Literature Analysis and Retrieval System Online; Lilacs = Literatura Latino-Americana e do Caribe em Ciências da Saúde; MeSH = Medical Subject Headings; DeCS = Descritores em Ciências da Saúde.

## CASE REPORT

A 68-year-old man came to the outpatient clinic reporting an electric shock-like sensation through his neck and spreading down his spine and lower limbs that was triggered by neck flexion. This was associated with paresthesia in his toes, gait disorders, progressive memory loss and slow thinking that had also been noticed over a three-month period preceding the appointment.

The examination on the patient revealed cognitive dysfunction (mini-mental state examination, MMSE = 18/30), glove and stocking hypoesthesia, proprioception loss in the lower limbs, wide-based gait and Romberg’s and Lhermitte’s signs. The cranial nerves, motor strength, muscle tone, coordination, muscle strength reflexes and plantar reflex were normal. Laboratory investigation revealed macrocystic anemia. The creatinine levels, liver function, blood electrolytes, coagulation profile and thyroid-stimulating hormone (TSH) were normal. The erythrocyte sedimentation rate (ESR) was 54 mm/h. The serum vitamin B12 level was 51 pg/ml (normal range = 200-900 pg/ml). The cerebrospinal fluid was normal with negative venereal disease research laboratory (VDRL) and anti-human immunodeficiency virus (anti-HIV) antibodies. A hiatus hernia and a gastric polyp were seen on gastroscopy. Histopathology was negative for malignancies. Nerve conduction evaluation revealed axonal sensory-motor peripheral polyneuropathy. Brain and spinal cord magnetic resonance imaging (MRI) were normal.

Intramuscular vitamin B12 therapy was started (5000 IU, monthly). Complete recovery was progressively achieved over a 12-month period.

## DISCUSSION

The neurological manifestations of vitamin B12 deficiency include peripheral and cranial nerve neuropathy and spinal cord and brain syndromes.^1^^,^^2^ Healton et al. found neurological signs in 74% of 143 vitamin B12 deficiency patients. Their findings were peripheral neuropathy (25%) myelopathy (12%), a combination of both of these (41%), neuropsychiatric dysfunction (8.1%) and visual deficits (0.5%).^1^ The presence of Lhermitte’s sign in patients with vitamin B12 deficiency has been previously described,^3^^,^^4^^,^^5^^,^^6^^,^^7^ and it is characterized by a sensation of electrical lightning discharge, with paresthesia experienced through the spine, legs and sometimes arms, caused by neck flexion.^8^

The pathophysiology of Lhermitte’s sign was classically described as related to the stretching of the hyperexcitable demyelinated dorsal column of the spinal cord, particularly at the cervical level, thereby triggering an electric shock-like sensation.^4^^,^^8^ Today, hyperexcitability is still regarded as the main pathophysiological mechanism. Moreover, from an etiological standpoint, in the original paper by Lhermitte, the shock-like sensations were attributed to medullar lesions due to demyelination or trauma of the dorsal column.^1^^,^^4^^,^^8^^,^^21^

Since then, although the presence of Lhermitte’s sign remains associated with such causes, particularly multiple sclerosis, several other causes have been described, including cervical disc hernia, cervical spondylosis, tumors, radiation myelopathy, herpes zoster, arachnoiditis, Behcet’s disease, atlantoaxial subluxation, and non-lesional disorders such as hyperexcitability of the ascending sensory neurons of the posterior column due to toxic causes.^4^^,^^8^^,^^22^

Increased T2-weighted and decreased T1-weighted signals, with contrast enhancement in the posterior and lateral columns of the spinal cord can be revealed by MRI.^21^^,^^22^^,^^23^

Lhermitte’s sign is rare in patients with vitamin B12 deficiency and may improve with specific therapy.
